# Liquid Phase Sintered Ceramic Bone Scaffolds by Combined Laser and Furnace

**DOI:** 10.3390/ijms150814574

**Published:** 2014-08-21

**Authors:** Pei Feng, Youwen Deng, Songlin Duan, Chengde Gao, Cijun Shuai, Shuping Peng

**Affiliations:** 1 State Key Laboratory of High Performance Complex Manufacturing, Central South University, Changsha 410083, China; E-Mails: fengpei@csu.edu.cn (P.F.); airyflier@csu.edu.cn (S.D.); gaochengde@csu.edu.cn (C.G.); 2 Department of Spine Surgery, the Second Xiangya Hospital of Central South University, Changsha 410011, China; E-Mail: drywdeng@163.com; 3 Shenzhen Research Institute, Central South University, Shenzhen 518057, China; 4 Cancer Research Institute, Central South University, Changsha 410078, China

**Keywords:** bone scaffolds, liquid phase, 45S5, mechanical properties, bioactivity

## Abstract

Fabrication of mechanically competent bioactive scaffolds is a great challenge in bone tissue engineering. In this paper, β-tricalcium phosphate (β-TCP) scaffolds were successfully fabricated by selective laser sintering combined with furnace sintering. Bioglass 45S5 was introduced in the process as liquid phase in order to improve the mechanical and biological properties. The results showed that sintering of β-TCP with the bioglass revealed some features of liquid phase sintering. The optimum amount of 45S5 was 5 wt %. At this point, the scaffolds were densified without defects. The fracture toughness, compressive strength and stiffness were 1.67 MPam^1/2^, 21.32 MPa and 264.32 MPa, respectively. Bone like apatite layer was formed and the stimulation for apatite formation was increased with increase in 45S5 content after soaking in simulated body fluid, which indicated that 45S5 could improve the bioactivity. Furthermore, MG-63 cells adhered and spread well, and proliferated with increase in the culture time.

## 1. Introduction

One of the major challenges in bone tissue engineering is the development of suitable scaffolds to repair bone defects. The scaffolds should be bioactive [1,2], with the ability to strongly bond with living bone tissues, and they should be biocompatible [3,4], promoting cellular interactions and bone tissue development. Moreover, they should have proper mechanical properties in order to maintain the shape [5,6]. Recently, β-TCP (β-Ca_3_(PO_4_)_2_) has been widely used as bone scaffold material due to good bioactivity, biocompatibility and osteoconduction [7,8,9]. However, the poor mechanical properties such as compressive strength and fracture toughness have severely restricted its use to low load-bearing applications [10,11]. Therefore, it is relevant to improve the strength and toughness, and further improve the bioactivity.

Generally, it has been believed that liquid phase sintering is an effective way to improve the mechanical properties of ceramics [12,13]. Hotta *et al.* [14] added aluminum nitride (AlN) as liquid phase into SiC ceramics and found that the strength increased about 1.16 times than that without AlN. Kalita *et al.* [15] added CaO-P_2_O_5_-Na_2_O based glass as liquid phase into hydroxyapatite ceramics and found that the hardness and strength increased from 3.2 GPa and 158 MPa to 4.6 GPa and 220 MPa as the glass content increased from 0–2.5 wt %, respectively. Hesaraki *et al.* [16] added 10 wt % of SiO_2_-CaO-MgO-P_2_O_5_ based glass as liquid phase into β-TCP and found that the strength and Vickers hardness were 1.32 and 1.26 times higher as compared to the pure β-TCP, respectively.

A SiO_2_-Na_2_O-CaO-P_2_O_5_ based bioglass, 45S5, whose chemical composition is close to that of the bone mineral phase, possesses excellent biocompatibility and osteoconductivity [17,18,19]. It can bond strongly to bone and promote bone growth by the formation of a bone like apatite layer [20,21]. More importantly, 45S5 can provide a liquid phase during the sintering of β-TCP due to the low melting point of 45S5 (about 1050 °C) compared with the sintering temperature of β-TCP (about 1100–1180 °C) [22,23]. The liquid phase may promote an additional diffusion mechanism of dissolution/precipitation, and affect particle rearrangement and capillary forces during the sintering process [24,25,26,27], which improves the densification and mechanical properties. In addition, 45S5 has unique bone bonding ability, which can provide faster response of the bone healing and bonding processes [28]. It can also release Si, Ca and P ions which enhance the adhesion, proliferation and differentiation of osteoblasts [29].

In this work, the β-TCP scaffolds were fabricated by selective laser sintering (SLS) combined with furnace sintering using 45S5 as liquid phase to improve the properties. The reasons were described as follows: SLS technique could fabricate the customized shapes and control the internal architectures of the porous scaffolds. However, it was difficult to obtain the high densities due to the short action time between the laser beam and materials during the SLS process. The use of furnace sintering after SLS could significantly improve the densities [30,31]. The sintering temperature was set at 1100 °C which was higher than the melting point of 45S5 (about 1050 °C) so that 45S5 could provide a liquid phase during the sintering process. In addition, the effects of 45S5 on the microstructure, phase composition, mechanical properties and bioactivity were investigated, and the cell’s response to the scaffolds was evaluated *in vitro*.

## 2. Results and Discussions

### 2.1. Porous Scaffolds

**Figure 1:**
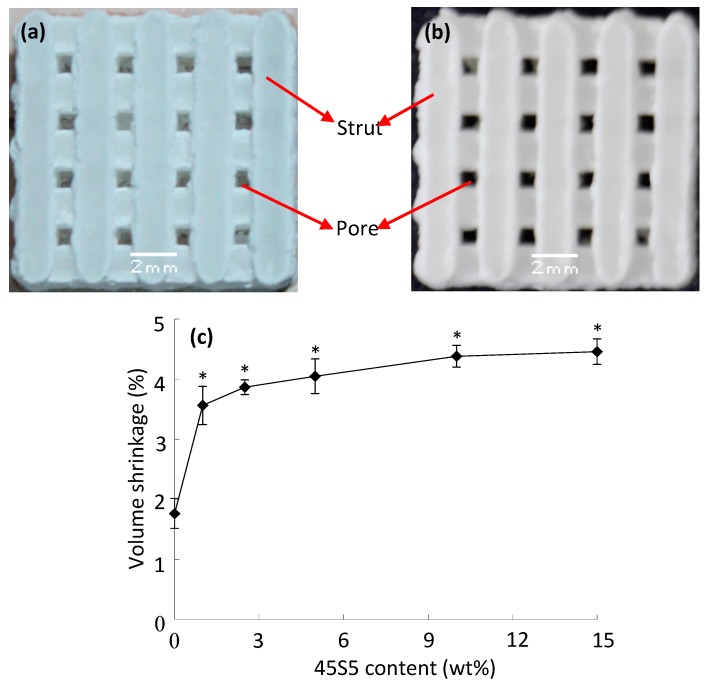
Scaffolds prepared by selective laser sintering (SLS) without (**a**) and with (**b**) furnace sintering at 1100 °C, and the effect of 45S5 content on the volume shrinkage of the scaffolds (**c**). Statistical analysis showed that the volume shrinkage of the scaffolds with 45S5 was significantly different from those without 45S5 (* *p* < 0.05) and there were significant differences between the scaffolds with different 45S5 content (* *p* < 0.05).

The fabricated scaffolds are shown in Figure 1a,b, respectively. The scaffolds have interconnected porous structures with well-controlled open pores. The sizes of the pores and struts are about 0.8 and 1.5 mm, respectively. It has been reported that pore size greater than ~300 μm is essential for vascularisation of constructs and bone ingrowth [32,33]. The effect of 45S5 content on the volume shrinkage of the scaffolds is shown in Figure 1c. The ratio of volume shrinkage increased sharply with an increase in 45S5 content from 0–1 wt %. Then it increased slightly to 4.46% with further increase in 45S5 content to 15 wt % and then was close to a constant. There were significant differences between the scaffolds with and without 45S5, and the scaffolds with different 45S5 content (*p* < 0.05). The reason was that the 45S5 particles melted into the liquid phase during the sintering process. The distance between the β-TCP particles decreased and consequently resulted in volume shrinkage.

### 2.2. Mechanical Properties

**Figure 2:**
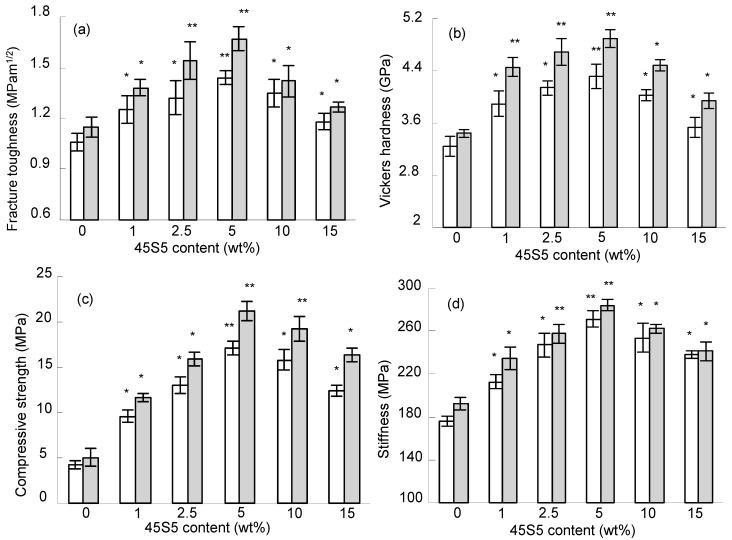
Fracture toughness (**a**); Vickers hardness (**b**); compressive strength (**c**) and stiffness (**d**) as a function of 45S5 content for the scaffolds fabricated by SLS without (□) and with (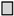) furnace sintering at 1100 °C. Statistical analysis showed that the mechanical properties of the scaffolds with 45S5 were significantly different from those without 45S5 (* *p* < 0.05, ** *p* < 0.01).

The fracture toughness, Vickers hardness, compressive strength and stiffness of the scaffolds are shown in Figure 2. The fracture toughness of the scaffolds with furnace sintering at 1100 °C increased sharply with an increase in 45S5 content from 0–5 wt % and reached a maximal value of 1.67 MPam^1/2^, which was 1.45 times higher than that of the scaffolds without 45S5. It slightly decreased to 1.27 MPam^1/2^ with further increase in 45S5 content up to 15 wt %. The trend of the Vickers hardness, compressive strength and stiffness of the scaffolds with 45S5 addition was similar to that of fracture toughness. The maximal Vickers hardness, compressive strength and stiffness were 4.89 GPa, 21.32 MPa and 264.32 MPa, which were 1.42, 4.16, 1.53 times higher than that of the scaffolds without 45S5, respectively. The mechanical properties of the scaffolds without furnace sintering were a little lower than that with furnace sintering. Additionally the mechanical properties of the scaffolds with 45S5 were significantly higher than those of the scaffolds without 45S5 (*p* < 0.05). Therefore, it could be concluded that the amount of 45S5 was 5 wt % for optimum mechanical properties.

### 2.3. Microstructure

**Figure 3:**
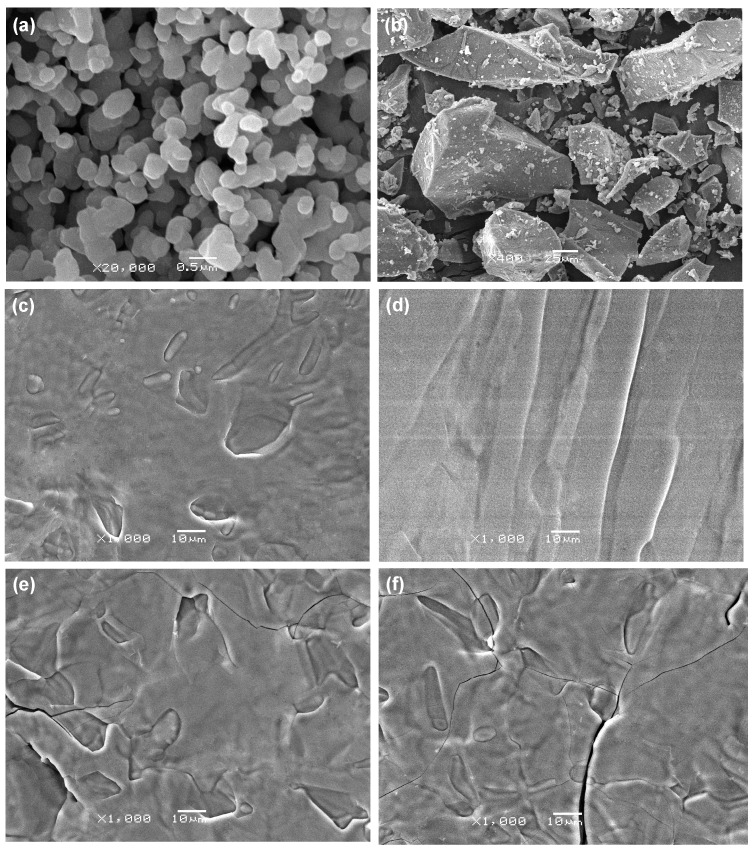
The raw powders (**a**,**b**) and the scaffolds with different 45S5 contents (**c**–**f**). β-tricalcium phosphate (β-TCP) (**a**); 45S5 (**b**); 1 wt % (**c**); 5 wt % (**d**); 10 wt % (**e**) and 15 wt % (**f**).

The raw β-TCP and 45S5 powders are shown in Figure 3a,b, respectively. The β-TCP powders exhibited spherical shape, and the average particle size was about 0.25 μm. The 45S5 powders had irregular shape and a wide distribution in particle sizes. The scaffolds with different 45S5 contents are shown in Figure 3c–f. A small amount of β-TCP particles were randomly dispersed (Figure 3c). The particles disappeared and there were no defects when the content of 45S5 was 5 wt % (Figure 3d), which indicated that 45S5 glassy liquid phase was well dispersed in the β-TCP ceramics matrix. However, some microcracks appeared when the content of 45S5 was 10 wt % (Figure 3e). The microcracks became enlarged and more numerous with further increase of 45S5 content to 15 wt % (Figure 3f). The emergence of microcracks might be due to the formation of a glassy continuous phase which led to a mismatch of the thermal expansion coefficient between β-TCP (13.1 × 10^−6^ K^−1^) and 45S5 (14.6 × 10^−6^ K^−1^) when the content was too high [34,35]. The microcracks were the reason for the decrease of mechanical properties.

**Figure 4:**
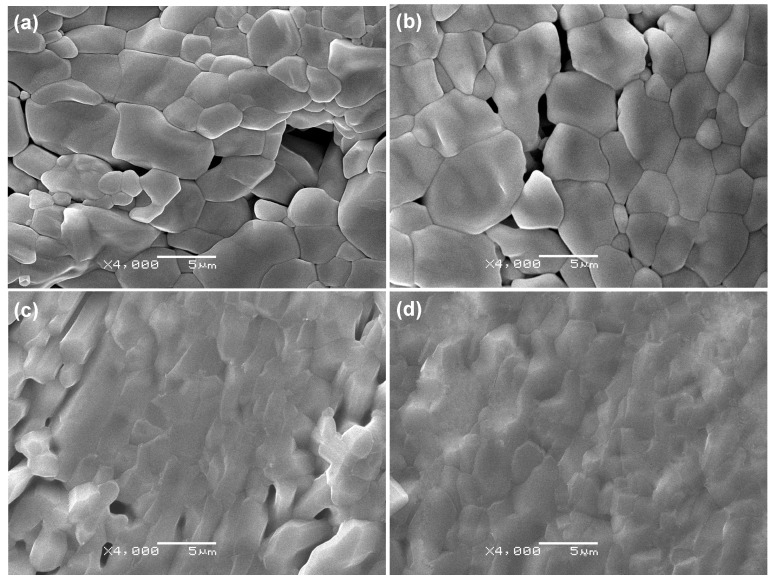
The polished and thermally etched scaffolds with different 45S5 content. 0 wt % (**a**), 1 wt % (**b**), 2.5 wt % (**c**) and 5 wt % (**d**).

The polished and thermally etched scaffolds with different 45S5 contents are shown in Figure 4. Some defects (e.g., pores) were observed and the densification of the scaffolds were poor when there was no 45S5 (Figure 4a). The defects became less and the densification was improved when 45S5 content was 1 wt % (Figure 4b). There were only a few small pores and the grain boundaries became illegible when the 45S5 content was 2.5 wt % (Figure 4c). The pores disappeared and the densification was significant improved when the 45S5 content was 5 wt % (Figure 4d). It indicates that liquid phase sintering was achieved in the scaffolds with 5 wt % 45S5 and the presence of a glassy liquid phase made the grain boundaries difficult to differentiate. The increase in densification caused by the glassy liquid phase was the reason for the increase of the mechanical properties when the 45S5 content increased from 0–5 wt %.

According to the sintering theory, there was only point contact between particles in the initial stage of sintering. However, as the temperature increased, on the one hand, the contact area between β-TCP particles increased. The particles aggregated and the volume shrunk under the action of surface energy, thus the densification increased. On the other hand, the 45S5 particles began to soften at 1050 °C and gradually melted into a glass liquid phase. The liquid phase took the gap as a channel under the action of high temperature and surface energy. Then, a layer of liquid membrane formed on the surface of the β-TCP particles which accelerated the particle movement and rearrangement. The volume of the scaffolds shrunk and the densification was further increased. However, microcracks emerged due to the mismatch of the thermal expansion coefficient which was caused by the formation of a glassy continuous phase when the content was too high.

Densification of the scaffolds increased sharply due to the increase of the glassy liquid phase with an increase in 45S5 content from 0–5 wt % as shown in Figure 1 and Figure 4. It was considered the primary reason for the increase of mechanical properties. However, no significant increment could be made on the densification by further increasing the 45S5 content, as shown in Figure 1. Moreover, the amount of microcracks increased due to the mismatch of the thermal expansion coefficient which was caused by the formation of a glassy continuous phase with further increase in 45S5 content to 15 wt % as shown in Figure 3. As a result, the mechanical properties were decreased.

### 2.4. Phase Composition

**Figure 5:**
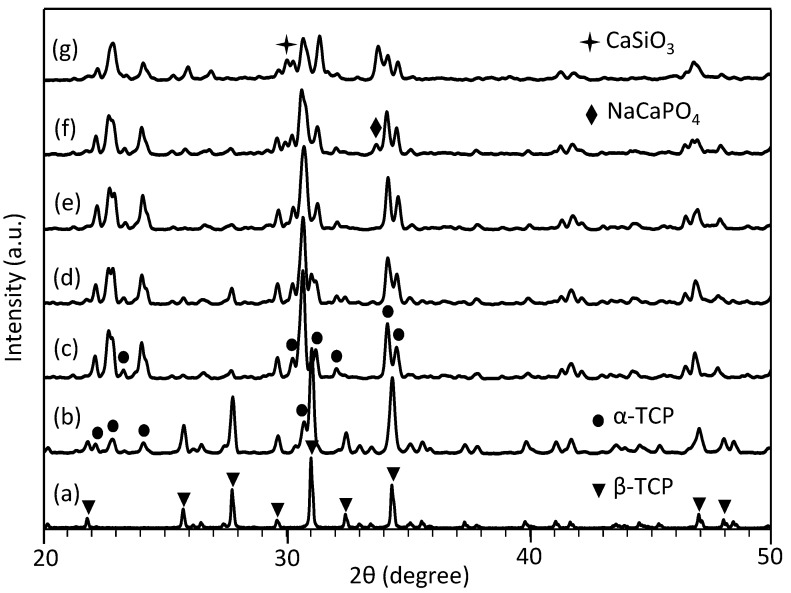
X-ray diffraction (XRD) patterns of raw β-tricalcium phosphate (β-TCP) powders (**a**) and scaffolds with different 45S5 contents (**b**–**g**). β-TCP (**a**); 0 wt % (**b**); 1 wt % (**c**); 2.5 wt % (**d**); 5 wt % (**e**); 10 wt % (**f**) and 15 wt % (**g**). ▼: β-TCP (JCPDF#09-0169), ●: α-TCP (JCPDF#09-0348), ♦: NaCaPO_4_ (JCPDF#29-1193) and 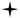: CaSiO_3_ (JCPDF#43-1460).

The XRD (X-ray diffraction) patterns of the raw β-TCP powders and the scaffolds with different 45S5 contents are shown in Figure 5. The diffractogram of the scaffolds presents the peaks ascribable to β-TCP (JCPDF#09-0169) and α-TCP (JCPDF#09-0348) when the 45S5 content was 5 wt % or below (Figure 5b–e). Two new phases, sodium calcium silicate (NaCaPO_4_, JCPDF#29-1193) and calcium silicate (CaSiO_3_, JCPDF#43-1460) were detected when the contents were 10 and 15 wt % (Figure 5f,g). It indicated that some reactions occurred during the sintering process and produced the above new phases.

**Figure 6:**
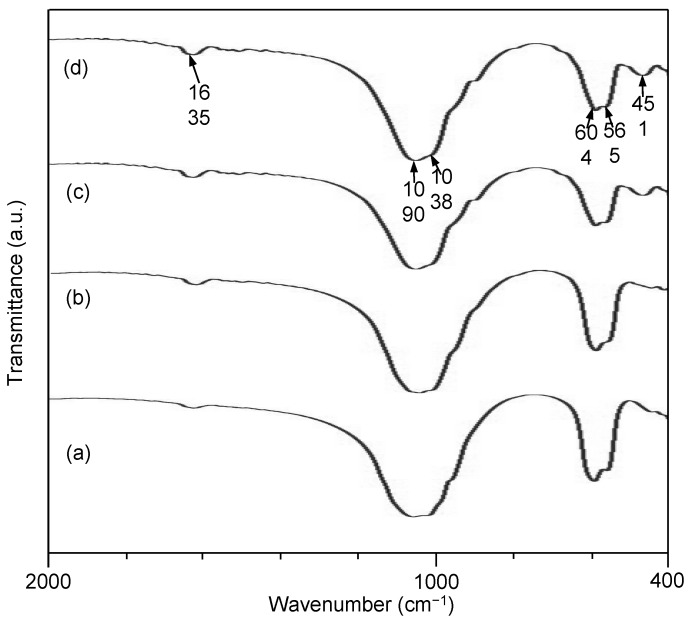
Fourier transform infrared spectroscopy (FTIR) spectra of the scaffolds with different 45S5 contents; 0 wt % (**a**), 1 wt % (**b**), 5 wt % (c) and 15 wt % (**d**).

FTIR (Fourier transform infrared spectroscopy) spectra of the scaffolds with different 45S5 contents are shown in Figure 6. The band at 1635 cm^−1^ was ascribed to absorbed water [36,37]. The band at 1090 cm^−1^ was attributed to the asymmetric stretching vibrations mode of phosphate groups [38], while the bands at 604 and 565 cm^−1^ were assigned to the bending vibration mode of phosphate groups [39,40]. The bands at 1038 and 451 cm^−1^ were assigned to the asymmetric and bending Si-O-Si stretching vibration of silicate groups, respectively [41,42]. The presence of Si-O-Si bending vibration which was attributed to normal vibration modes of Si-O in the SiO_4_ group, indicated the presence of amorphous silicate [43]. The two independent peaks at 1090 and 1038 cm^−1^ imply the occurrence of a glassy phase separation, namely, the appearance of both a silicate- and a phosphate-rich phase [44,45]. The intensity of the silicate absorption bands at 474 cm^−1^ increased as 45S5 content increased.

### 2.5. In Vitro Characterization

**Figure 7:**
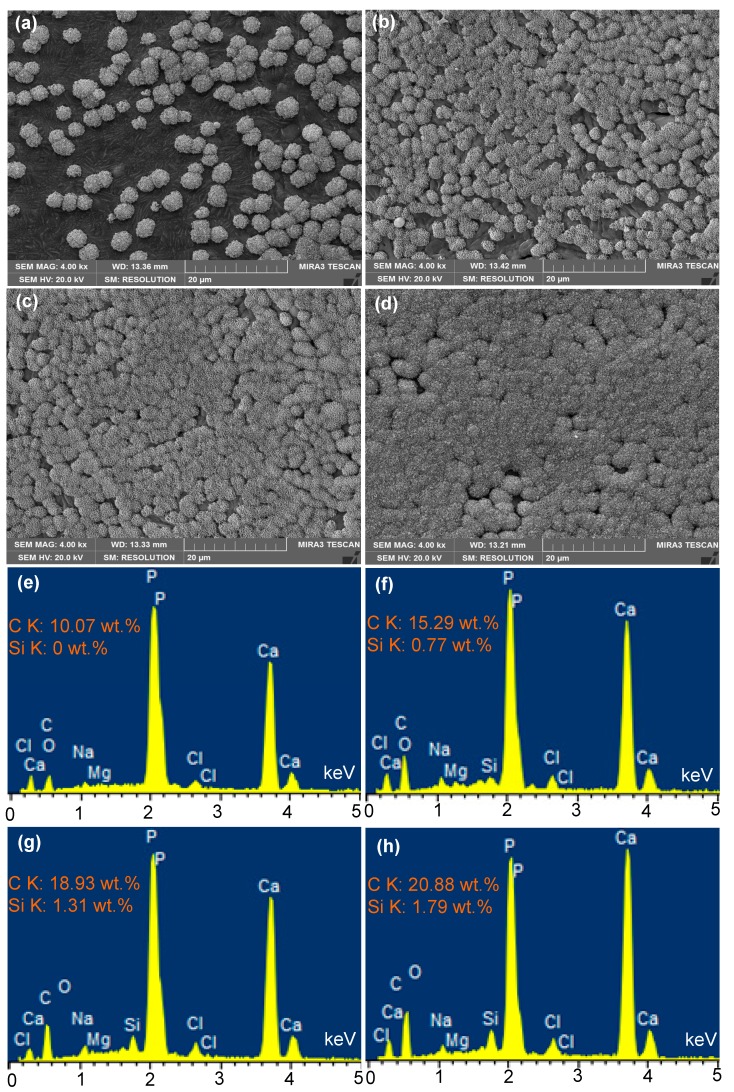
Scanning electron microscopy (SEM) micrographs and energy dispersive X-ray spectrometry (EDS) analysis of the scaffolds with different 45S5 contents after soaking in Simulated body fluid (SBF) for 14 days; 0 wt % (**a**,**e**), 2.5 wt % (**b**,**f**), 5 wt % (**c**,**g**) and 15 wt % (**d**,**h**).

The scaffolds with different 45S5 contenst soaked in Simulated body fluid (SBF) for 14 days are shown in Figure 7. It could be seen that some tiny aggregates of the apatite appeared on the scaffolds without 45S5 after soaking in SBF (Figure 7a). The apatite increased when the 45S5 content was 2.5 wt % (Figure 7b). A homogeneous apatite layer was formed on the whole scaffolds surface when the 45S5 content increased to 5 wt % or above (Figure 7c,d). The energy dispersive X-ray spectrometry (EDS) results showed that the apatite on the surface of scaffolds without 45S5 consisted of a calcium phosphate, and it contained no Si but did have some C, Na, Mg and Cl. The apatite on the surface of scaffolds with 45S5 consisted of a calcium phosphate with Si and some C, Na, Mg and Cl. Moreover, the content of Si and C increased with increase of 45S5 content. The presence of C element in the apatite indicated the formation of hydroxy-carbonate-apatite (HCA).

This indicated that the addition of 45S5 could improve the apatite-forming ability of scaffolds. The breakage of Si-O-Si bonds and the formation of silanols (Si-OH) at the glass-solution interface led to the formation and growth of an amorphous CaO-P_2_O_5_-rich film, which provided favorable sites for nucleation of apatite crystals [46,47]. The solubility of the β-TCP resulted in a supersaturation of Ca^2+^ and PO_4_^3−^ ions. The OH^−^ and CO_3_^2−^ ions from the SBF solution incorporated with Ca^2+^ and PO_4_^3−^ ions to form a mixed HCA. The composite scaffolds provided more nucleation sites for the apatite crystals as the content of 45S5 increased.

**Figure 8:**
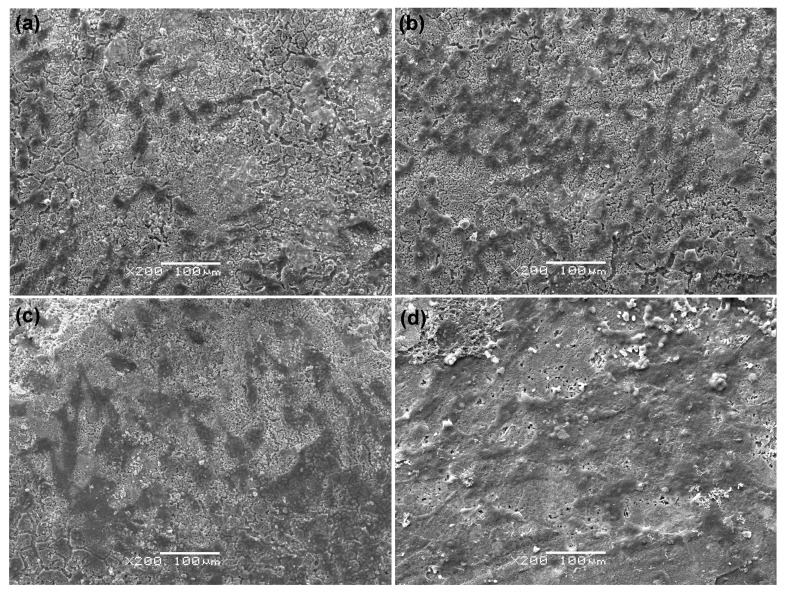
The morphological features of cells cultured on the scaffolds with 5 wt % 45S5 for 4 h (**a**); 1 day (**b**); 3 days (**c**) and 5 days (**d**).

The MG-63 cells attachment on the scaffolds with 5 wt % 45S5 are shown in Figure 8. MG-63 cells firmly attached and spread well on the surface of the scaffolds after 4 h of culture (Figure 8a). The numbers of cells significantly increased after one day of culture (Figure 8b). They were spread out and exhibited an elongated morphology with the formation of extended filopodia after three days of culture (Figure 8c). The cells covered the whole scaffolds and cell-to-cell junctions appeared after five days of culture (Figure 8d). The results showed an increase in the levels of proliferation as a function of incubation time. The scaffold had a positive effect on osteoblast cells response in terms of adhesion and proliferation. There are two main factors that might affect MG-63 cells behavior on the scaffolds. One is the Si ions released from scaffolds which might stimulate cell adherence and proliferation [48]. The second is the bone like apatite layer on the scaffolds surface which might benefit cell spreading and proliferation [49].

## 3. Experimental Section

### 3.1. Preparation of the Composite Powders

The β-TCP powders (Kunshan Chinese Technology New Materials Co., Ltd., Kunshan, China), with average particle size of 0.1–0.3 µm, were prepared by a co-precipitation method using Ca_3_(PO_4_)_2_ and (NH_4_)_2_HPO_4_. The 45S5 (wt %: 45% SiO_2_, 24.5% Na_2_O, 24.5% CaO and 6% P_2_O_5_) powders (Kunshan Chinese Technology New Materials Co., Ltd.), with average particle size below 48 μm, were prepared by melting the raw materials (commercial SiO_2_, Ca_3_(PO_4_)_2_, Na_2_CO_3_ and CaCO_3_). The composite powders were prepared by adding 45S5 powders (1, 2.5, 5, 10 and 15 wt %) to the β-TCP powders. They were mixed for 1 h using a mechanical mixing method.

### 3.2. Fabrication and Characterization of β-Tricalcium phosphate (β-TCP) Scaffolds

Firstly, the β-TCP scaffolds were fabricated using the home-made SLS system. The details of the laser sintering system have been reported previously [50]. The following processing parameters were used: spot diameter of 1.2 mm, laser power of 7.8 W, scan speed of 120 mm/min and layer thickness of 0.1–0.2 mm. Then, the scaffolds were put into a furnace (JNL-16XB, Luoyang Liyu Co., LTD., Luoyang, China) and sintered according to the follows process. The temperature was slowly heat to 1100 °C at a heating rate of 0.5 °C/min and kept for 3 h. Subsequently, it was cooled to 400 °C at a cooling rate of 0.5 °C/min and then slowly cooled to room temperature with the furnace.

The morphologies were observed by scanning electron microscopy (SEM) (JEOL JSM-5600LV, JEOL Ltd., Tokyo, Japan). The scaffolds were thermally etched at 1050 °C for 20 min in order to delineate the grain boundaries. The phase compositions were examined by X-ray diffraction (XRD) (D8-ADVANCE, Bruke, Germany) with Cu Kα radiation (λ = 1.5418 Å). The diffractometer was operated at 40 kV and 40 mA over a 2θ range of 20–50 degrees at a step size of 0.02 degree and a scanning speed of 8 degree/min. The peaks obtained were compared to standard reference Joint Committee on Powder Diffraction and Standards (JCPDS) files. The functional groups were determined by Fourier transform infrared spectroscopy (FTIR) (NicoletteTM 6700, Thermo Electron Corp., Madison, WI, USA) in the range of 2000–400 cm^−1^.

The volume shrinkage (Δ_%_) was calculated according to the equation [51]: Δ_%_ = (*d*_S_ − *d*_F_)·100/*d*_S_, where *d*_S_ is the volume of the scaffolds without furnace sintering, *d*_F_ is the volume of the scaffolds with furnace sintering. The Vickers hardness was measured using a Vickers microindenter (HXD-1000TM/LCD, Digital Micro Hardness Tester, Shanghai Taiming Optical Instrument Co., Ltd., Shanghai, China). The applied load used for each indentation was set at 300 gf with a dwell time of 15 s. The fracture toughness (*K_IC_*) was calculated according to the Evans-Charles formula [52]: *K_IC_* = 0.0824*PC*^−3/2^, where *P* is the applied indentation load and *C* is the length of the induced radial crack. Compressive properties of the scaffolds (12 × 12 × 8 mm^3^) were determined using a microcomputer control electronic universal test machine (WD-D1, Shanghai Zhuoji instruments Co., Ltd., Shanghai, China) at a crosshead speed of 0.5 mm/min. The compressive strength was determined from the first point on the stress-strain curve at which an increase in strain was observed without an increase in stress. The stiffness was determined from the initial linear region of compressive stress-strain curve.

### 3.3. Bioactivity in Simulated Body Fluid (SBF)

The bioactivity of the scaffolds was evaluated by examining the formation of bone like apatite on the surface in the SBF, which was prepared as previously described by Kokubo [53] and had similar ion concentrations and pH value to those of human blood plasma (HBP) (Table 1). The scaffolds were soaked in the SBF solution for 14 days at 37 °C with a ratio of mass to solution volume of 0.15 g/30.0 mL. The solution was refreshed every day. The scaffolds were removed from the SBF, gently rinsed with distilled water, and then dried in an electrothermal blowing dry box (101-00S, Guangzhou Dayang Electronic Machinery Equipment Co., Ltd., Guangzhou, China) before further characterization. The formation of bone like apatite was characterized by energy dispersive X-ray spectrometry (EDS, X-Max 20 mm^2^, Oxford Instruments, Oxford, UK) and the morphology was observed using SEM (Tescan, Mira 3 FEG-SEM, TESCAN Co., Brno, Czech).

**Table 1:** Comparison of ion species and concentration between simulated body fluid (SBF) and human blood plasma (HBP) (mmol/L).

Ion Species	SBF	HBP
Na^+^	142.0	142.0
K^+^	5.0	5.0
Mg^2+^	1.5	1.5
Ca^2+^	2.5	2.5
Cl^−^	148.8	103.0
HCO_3_^−^	4.2	27.0
HPO_4_^2−^	1.0	1.0
SO_4_^2−^	0.5	0.5

### 3.4. Cell Attachment and Proliferation

Human osteoblast-like MG-63 cells were obtained from American Type Culture Collection (ATCC, Rockville, MD, USA). The cells were cultured and proliferated in a cell culture plate for 1 day prior to seeding. And then cultured in Dulbecco’s modified Eagle’s medium (DMEM, Gibco, Carlsbad, CA, USA) supplemented with 5% penicillin/streptomycin antibiotics (Sigma, St. Louis, MO, USA) and 10% fetal bovine serum (FBS), incubated at 37 °C and 5% CO_2_. The medium was changed every 2 days. Cell subcultures at passage two were used in the following studies. The scaffolds were sterilized by immersion in 75% ethanol for 10 min. After sterilization, the scaffolds were washed and immersed in phosphate-buffered saline (PBS) for 1 day. Then 2 × 10^5^ cells/mL were added to each scaffold and allowed to attach to the matrix for 30 min before adding enough medium to submerge the scaffolds. Cell culture was allowed to continue for 4 h, 1 day, 3 and 5 days in a humidified atmosphere of 5% CO_2_ at 37 °C. At a pre-selected time point, the scaffold-cell constructs were removed, rinsed with PBS twice and fixed with 2.5% glutaraldehyde for 30 min. The fixed constructs were washed with PBS three times, dehydrated in graded ethanol, dried at 37 °C overnight and sputter-coated with gold-palladium prior to SEM observation.

### 3.5. Statistical Analysis

Experimental data were expressed as means ± SD (Standard Deviation). Statistical difference was analyzed using SPSS software (SPSS Statistics version 19, IBM, Armonk, NY, USA). The *p* value of less than 0.05 or 0.01 was considered significant or very significant, respectively.

## 4. Conclusions

β-TCP scaffolds with different amounts of 45S5 were fabricated by SLS combined with furnace sintering using 45S5 as liquid phase to improve the properties. The mechanical properties increased with increase in 45S5 content from 0–5 wt % and then decreased with further increase in 45S5 content up to 15 wt %. The maximum fracture toughness, Vickers hardness, compressive strength and stiffness values were 1.67 MPam^1/2^, 4.89 GPa, 21.32 MPa and 264.32 MPa, respectively. The increase in mechanical properties was attributed to the increase in densification caused by introducing of the glassy liquid phase. The decrease was due to the emergence of microcracks caused by formation of a glassy continuous phase when the content of 45S5 was too high. The ability to form a bone like apatite layer on the scaffolds in SBF increased with increase in 45S5 content, indicating 45S5 could improve the bioactivity. Furthermore, cells adhered and spread well on the scaffolds, indicating favorable cytocompatibility. In conclusion, the scaffolds with 5 wt % 45S5 exhibit promising prospects for bone regeneration.
